# Supplementary material for: Germline Genetic Mutations in Adult Patients with Sarcoma: Insight into the Middle East Genetic Landscape
Source: Cancers (Basel). 2024 Apr 25;16(9):1668. doi: 10.3390/cancers16091668 (PMC11083501; doi:10.3390/cancers16091668)
Supplement: Supplementary file 1 [file cancers-16-01668-s001.zip › Table Suppl 2.pdf]

Supplementary Table S2: Details of pathogenic/likely pathogenic germline mutations:

Genes	Types	Exon/ Intron	Nucleotide Changes	Amino Acid Change	Variant Type	Gender	Stage	Location	Family History	2nd Primary tumors
APC	Increased Risk Allele	Exon 16	c.3920T>A	p.Ile1307Lys	Missense	Female	Localized	Lower Limp	Positive	None
APC	Increased Risk Allele	Exon 16	c.3920T>A	p.Ile1307Lys	Missense	Male	Localized	Lower Limp	Positive	None
APC	Increased Risk Allele	Exon 16	c.3920T>A	p.Ile1307Lys	Missense	Female	Localized	Lower Limp	Positive	None
APC	Increased Risk Allele	Exon 16	c.3920T>A	p.Ile1307Lys	Missense	Female	Metastatic	Upper limp	Negative	None
APC	Pathogenic	Exon 16	c.4393_4394del	p.Ser1465Trpfs*3	Deletion	Female	Localized	Lower Limp	Positive	FAP
BRCA1	Pathogenic	Exon 11	c.4117G>T	p.Glu1373*	Nonsense	Female	Localized	Upper limp	Positive	None
BRCA2	Pathogenic	Exon 11	c.5351dup	p.Asn1784Lysfs*3	Duplication	Female	Localized	Upper limp	Positive	Breast
BRCA2	Pathogenic	Exon 11	c.2254_2257del	p.Asp752Phefs*19	Deletion	Female	Localized	Upper limp	Positive	Breast
BRIP1	Likely Pathogenic	Exon 20	c.3260dup	p.Asn1087Lysfs*4	Duplication	Female	Localized	Upper limp	Negative	None
BRIP1	Pathogenic	Exon 9	c.1315C>T	p.Arg439*	Nonsense	Female	Localized	Upper limp	Positive	Breast
CDKN2A	Likely Pathogenic	Exon 1	c.172C>T	p.Gln58*	Nonsense	Male	Localized	Upper limp	Positive	None

CHEK2	Pathogenic	Exon 4	c.470T>C	p.Ile157Thr	Missense	Male	N/A	Upper limp	Positive	None
MUTYH	Pathogenic	Exon 7	c.545G>A	p.Arg182His	Missense	Male	Metastatic	Lower Limp	Positive	Colorectal
NF1	Pathogenic	Exons 9-35	Deletion	Deletion	Deletion	Female	Localized	Upper limp	Positive	None
NF1	Pathogenic	Exon 13	c.1466A>G	(p.Tyr489Cys)	Missense	Female	Localized	Upper limp	Negative	Breast
NF1	Pathogenic	Deletion of the Entire coding sequence	Deletion of the Entire coding sequence	Deletion of the Entire coding sequence	Deletion	Female	Metastatic	Lower Limp	Positive	None
PALB2	Pathogenic	Exon 4	c.487_488del	p.Val163Leufs*4	Deletion	Female	Localized	Upper limp	Negative	Lymphoma
TP53	Pathogenic	Exon 5	c.422G>A	p.Cys141Tyr	Missense	Female	localized	Upper limp	Positive	Breast
TP53	Pathogenic	Exon 5	c.541C>T	p.Arg181Cys	Missense	Female	Metastatic	Lower Limp	Positive	None
TP53	Pathogenic	Exon 5	c.427G>A	p.Val143Met	Missense	Male	Metastatic	Upper limp	Positive	None
